# Extraction and Characterization of Starches from Varieties of Oca (*Oxalis tuberosa*), a High-Andean Tuber

**DOI:** 10.3390/polym18081000

**Published:** 2026-04-21

**Authors:** Caterine Pariona-Gutiérrez, David Choque-Quispe, Fredy Taipe-Pardo, Carlos A. Ligarda-Samanez, Diego E. Peralta-Guevara, Jeny Pariona-Gutiérrez, Jhoel Flores-Alvarez, Jakelin Pariona Gutiérrez

**Affiliations:** Department of Agroindustrial Engineering Academic, José María Arguedas National University, Andahuaylas 03701, Peru; ftaipe@unajma.edu.pe (F.T.-P.); caligarda@unajma.edu.pe (C.A.L.-S.); deperalta@unajma.edu.pe (D.E.P.-G.); jenyparionagutierrez@gmail.com (J.P.-G.); jflores@unajma.edu.pe (J.F.-A.); jakelinparionag10@gmail.com (J.P.G.)

**Keywords:** *Oxalis tuberosa* (oca), starch, thermal behavior, functional behavior

## Abstract

*Oxalis tuberosa* (Oca) is traditionally cultivated in the high Andean regions of Peru and represents a promising alternative source of starch with potential industrial uses, ranking among the most essential tubers after the potato. This study aimed to evaluate the physicochemical, morphological, techno-functional, and thermal properties of starch isolated from three specific varieties of Oca (yellow, black, and white) harvested at the Ccanccayllo production center in Andahuaylas, Peru. The isolated starches exhibited high purity, characterized by high luminosity (L* > 92.28) and a whiteness index exceeding 92.10. Moisture content ranged from 9.36% to 10.01%, correlating with low water activity (a_w = 0.44), indicating stability. Notably, the amylose content was significantly higher than that of other previously studied Oca varieties. This composition contributed to a favorable water absorption capacity, solubility index, swelling power, and viscosity, with the white variety displaying superior functional performance. Colloidal stability in aqueous media was moderate, as indicated by zeta potential analysis. Particle size analysis revealed granules ranging from 26.32 to 27.74 μm, with elongated and oval morphologies confirmed by SEM, displaying characteristic functional groups. Thermal analysis (DSC) demonstrated gelatinization temperatures between 52.73 and 53.12 °C and enthalpies ranging from 4.92 to 6.11 J/g, while Thermogravimetric Analysis (TGA) indicated thermal degradation up to approximately 74–80%. These findings suggest that the studied Oca starches possess significant potential for application in the food and pharmaceutical industries due to their distinct functional properties.

## 1. Introduction

Oca is a tuber native to the central Andes, traditionally cultivated at altitudes exceeding 3000 m, especially in rural regions of Peru and Bolivia. Since pre-Incan times, this crop has been an essential component of Andean community diets, not only as a carbohydrate source but also for its notable resilience to adverse climatic conditions. Unlike other crops, oca tolerates low-fertility soils, scarce precipitation, and low temperatures, making it a viable alternative in the face of climate change and soil degradation [1]. However, despite its cultural and agricultural importance, oca remains an underutilized crop with limited technological valorization.

One of the main components of oca is starch, a reserve polysaccharide that serves as a primary energy source in the human diet. This biopolymer consists of two macromolecules: amylose, a linear macromolecule, and amylopectin, a highly branched macromolecule. The ratio between them directly influences physicochemical and functional properties such as viscosity, gelatinization, gel formation, swelling capacity, and retrogradation [2,3]. Due to these characteristics, starch is used in the food industry as a thickener, stabilizer, and texturizing agent. It is also employed in non-food sectors such as pharmaceuticals, cosmetics, paper, textiles, and the production of biodegradable materials.

However, native starches, including those from conventional sources such as corn, potato, or rice, have limitations that limit their use in demanding industrial conditions. Key disadvantages include low thermal stability, sensitivity to acidity and freeze–thaw cycles, limited shear resistance, and a tendency to retrograde during storage [4,5]. Furthermore, many starches exhibit poor cold-water solubility and limited functionality in complex food matrices [6]. These shortcomings have sparked interest in studying new starch sources, such as oca, which may offer more favorable functional properties than those of alternative or underexplored crops.

Oca starch represents an interesting and underexplored alternative. Previous studies have suggested that starches derived from lesser-known Andean tubers could present distinct properties, such as higher swelling power, lower gelatinization temperatures, or a favorable rheological profile for certain processes [7,8,9]. Although various studies on oca starch properties exist, further characterization of different varieties is needed, considering genetic and environmental differences, to establish their application potential in different industrial sectors.

Starch functionality depends on various factors, including granular structure, particle size, crystallinity, moisture content, and amylose/amylopectin ratio [10]. These attributes influence key parameters, including water absorption capacity, peak viscosity, gelatinization temperature, thermal stability, and retrogradation. Evaluating these properties in starches from local oca varieties not only enhances understanding of their processing behavior but also promotes the revalorization of native crops that have been historically marginalized in scientific research and industrial development.

Therefore, this study aimed to characterize starch extracted from three oca (*Oxalis tuberosa*) varieties cultivated in the Peruvian highlands by evaluating their physicochemical, structural, thermal, and techno-functional properties. The results will help establish their potential use as an ingredient in food and non-food applications, thereby contributing to the sustainable utilization of native agrobiodiversity resources with high strategic value.

## 2. Materials and Methods

### 2.1. Raw Material

**Figure 1:**
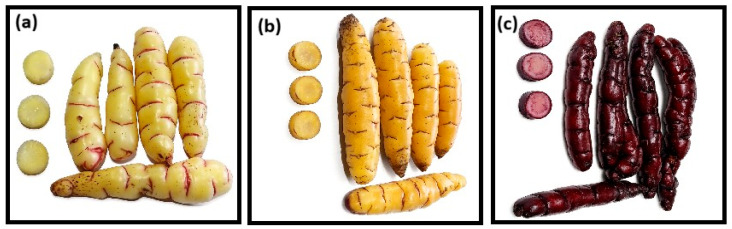
Varieties of oca: (**a**) white, (**b**) yellow, and (**c**) black.

Oca tubers of white (WOS), yellow (YOS) and black (BOS) varieties (Figure 1), cultivated during the 2023–2024 harvest period, were collected at physiological maturity post-flowering in June in the Ccanccayllo community (13°44′55.9″ S, 73°30′37.2″ W, 3433 m of altitude), Andahuaylas, Peru.

### 2.2. Starch Extraction

Tubers were thoroughly washed to remove adhering soil and debris, then cut into approximately 2 cm cubes and homogenized at maximum speed using a domestic blender. The slurry was filtered through an 80-mesh sieve, and the filtrate was decanted and allowed to settle for 24 h. The supernatant was carefully discarded, and the starch sediment was recovered and washed with distilled water by centrifugation at 6000 rpm for 5 min at 6 °C. The wet starch was dried in a forced-air oven (UF160mplus, Memmert, Büchenbach, Germany) at 45 °C for 24 h, milled, passed through a 65-mesh sieve, and stored in containers until analysis [11].

### 2.3. Yield Determination

Starch extraction yield was determined using Equation (1) [12].

(1) $$\%EY=\frac{Wes}{Wo}*100$$

where % EY is the extraction yield percentage; Wes is the weight of extracted starch; and Wo is the initial weight of oca tubers.

### 2.4. Color

Color was determined using a colorimeter (Model CR-5, Konica-Minolta, Tokyo, Japan) in the L*, a*, b* color space. The whiteness index (WI), yellow index (YI), and color index (CI) were calculated using Equations (2)–(4), respectively [13].

(2) $$WI=100-\sqrt{{\left(100-L^{*}\right)}^{2}+{\left(a^{*}\right)}^{2}+{\left(b^{*}\right)}^{2}}$$

(3) $$YI=\frac{142.86\times b^{*}}{L^{*}}$$

(4) $$CI=\frac{\left(a^{*}\times 1000\right)}{L^{*}\times b^{*}}$$

### 2.5. Moisture Content and Water Activity

Moisture content was determined following the AOAC 964.22 standard method AOAC. Water activity was measured using a calibrated instrument (Model HygroPalm23-AW, Rotronic, Bassersdorf, Switzerland) following the manufacturer’s protocols.

### 2.6. Zeta Potential and Particle Size

Analysis was performed using a Zetasizer ZSU3100 (Malvern Instrument, Malvern, UK). Starch (0.15 g) was mixed with 50 mL of ultrapure water and vortexed at 3500 rpm. The mixture was placed in a disposable folded capillary cell DTS 1080. Measurements were taken at 25 °C with a wavelength of 632.8 nm.

For particle size, 2 g of starch was mixed in 250 mL of isopropyl alcohol (99.5%), sonicated for 60 s at 30% amplitude, and then analyzed using a Dynamic Light Scattering (DLS) instrument (Mastersizer 3000, Malvern Instruments, Malvern, UK) with continuous stirring at 1500 rpm for 10 s.

### 2.7. Morphological Determination

Starch samples were fixed on carbon tape and examined using a Quanta 200 scanning electron microscope (SEM, Thermo Fisher, Waltham, MA, USA) under low vacuum at 25 kV acceleration and 1000× magnification.

### 2.8. XRD Analysis

Starch samples were analyzed using a Bruker D8-Focus diffractometer (Karlsruhe, Germany) with a Cu Kα1 radiation source (λ = 1.5406 Å) at 40 kV and 40 mA, equipped with a Lynxeye PSD-type detector. Scans were performed over a 5° to 60° (2θ) angular range with a step size of 0.04°.

The degree of crystallinity (DC) was calculated as the ratio of the area corresponding to crystalline regions to the total area of the diffractogram, using Equation (5) [14].

(5) $$DC\;(\%)=\frac{Sct.p}{St}*100$$

where Sct.p is the area of the crystalline phase, and St is the total area of the diffractogram.

### 2.9. Amylose and Amylopectin Content Determination

Starch samples (10 mg) were mixed with 0.1 mL of 95% ethanol and 0.9 mL of 1 M sodium hydroxide, and left at ambient temperature for 24 h. The volume was then adjusted to 10 mL with ultrapure water, vortexed rigorously, and left to stand for 30 min. An aliquot (0.5 mL) was mixed with 1 mL of 1 M acetic acid, followed by 0.2 mL of Lugol’s solution. The volume was adjusted to 10 mL with deionized water, and the mixture was kept in darkness for 20 min. Absorbance was measured at 620 nm using a UV-Vis spectrophotometer [15]. A calibration curve was prepared using a 1 mg/mL solution of amylose in deionized water. Amylose content (%) was calculated using Equation (6).

(6) $$\%\;Amylose=\frac{M*d}{f}*100$$

where M is the amylose mass (mg) from the calibration curve, d is the dilution factor, and f is the starch mass. Amylopectin content was determined by calculating the difference.

### 2.10. IR Analysis

Starch samples were prepared as 0.1% KBr pellets and analyzed using an FTIR spectrometer (Nicolet IS50, Thermo Fisher, Waltham, MA, USA) in transmission mode. Spectra were recorded in the range of 4000 to 400 cm^−1^ with a resolution of 4 cm^−1^.

### 2.11. Determination of Water Absorption Capacity (WAC), Water Solubility Index (WSI), and Swelling Power (SP)

A starch suspension (0.2 g in 15 mL distilled water) was heated in a water bath at controlled temperatures ranging from 50 °C to 90 °C for 30 min with constant stirring. After cooling to room temperature, the suspension was centrifuged at 2500 rpm for 30 min. The supernatant was decanted and dried in Petri dishes at 90 °C for 24 h. The weight of the swollen starch gel sediment and the dried supernatant was recorded [16]. The indices were calculated using Equations (7)–(9).

(7) $$WAC=\frac{weight\;of\;gel\;(g)}{weight\;of\;sample\;(g)}$$

(8) $$WSI=\frac{weight\;of\;dried\;supernatant\;(g)}{weight\;of\;sample\;(g)}*100$$

(9) $$SP=\frac{weight\;of\;gel\;(g)}{weight\;of\;sample\;\left(g\right)-weight\;of\;dried\;supernatant\;(g)}$$

### 2.12. Rheological Behavior Determination

Flow curves were obtained for 5% (*w*/*v*) starch suspensions using a rotational rheometer (Anton Paar MCR702e, Graz, Austria) with a concentric cylinder geometry. Measurements were performed under a controlled shear rate from 1 to 30 s^−1^. The experimental data were fitted to the Power Law model for non-Newtonian fluids (Equation (10)).

(10) $$\tau =k\gamma^{n}$$

where τ is the shear stress (Pa); γ is the shear rate (s^−1^); k is the consistency index (Pa·s^n^); and n is the flow behavior index.

### 2.13. Thermal Analysis

Starch thermal stability was determined through Thermogravimetric Analysis (TGA). An amount of 10 milligrams of starch was analyzed using TA Instruments equipment, model TGA550 (New Castle, DE, USA), over a temperature range of 20 to 600 °C at a heating rate of 10 °C/min under a nitrogen (N_2_) atmosphere [17].

Thermal transition properties were analyzed using a Differential Scanning Calorimeter (DSC2500, TA Instruments, New Castle, DE, USA) under a nitrogen flow rate of 50 mL/min. Samples were prepared at a 1:2 starch-to-water ratio, sealed in aluminum pans, and heated from 20 to 200 °C at 5 °C/min [18].

### 2.14. Statistical Analysis

Physical, chemical, techno-functional, and thermal properties were evaluated using a Completely Randomized Design with three replicates. Analysis of variance (ANOVA) was performed at a 5% significance level; when significant differences were found, Tukey’s mean comparison test was applied. Data were processed using STATGRAPHICS^®^ CENTURION XVI, and graphs were generated using Origin Pro 2023.

## 3. Results and Discussion

### 3.1. Yield, Color, Moisture Content, Water Activity, Particle Size, and Zeta Potential

Starch yields are shown in Table 1, with significant differences (*p* < 0.05) observed. The highest yield was for the WOS variety (8.25%), and the lowest for the YOS variety (7.58%). Differences in starch content among oca varieties are attributed to climatic conditions, soil type, tuber maturity, extraction methods and varietal growth conditions [19,20].

The starch yield obtained in this study (~8%) is relatively low compared with conventional starch sources, which could limit its economic feasibility for large-scale production. However, oca starch exhibits distinctive physicochemical and functional properties that could justify its use in specific or value-added applications, where the functional performance of the starch is more relevant than the extraction yield.

Color is an important attribute influencing consumer acceptability and final product quality. The color parameters (L*, a*, b*) and whiteness indices for oca starches are presented in Table 1, showing significant differences (*p* < 0.05). Luminosity (L) values exceeded 92.28, and whiteness indices exceeded 92.10. L* values greater than 90 indicate high-purity starches [21], confirming the near-white appearance of oca starch. Chroma a* values were near zero for all varieties, indicating a neutral color within the a* range [22], while low b* values suggest a slight yellowish tendency [23]. Differences among varieties are likely due to pigment leaching, such as anthocyanins in black oca and carotenoids in the yellow variety [24].

Similarly, yellow index (YI) and color index (CI) values complement this characterization. The BOS variety showed the highest YI, indicating a greater contribution of yellow pigments, possibly from oxidized anthocyanins or retained carotenoids [25]. In contrast, WOS exhibited the lowest YI, suggesting higher whiteness and less interference from colored compounds. Regarding CI values, WOS showed the highest (2.43), indicating greater chromatic intensity, while the negative CI value for BOS (−0.25) suggests a more neutral color, consistent with pigment loss during extraction [26,27].

Moisture content and a_w_ are fundamental parameters for the stability, storage, and quality of starch, as they describe the state and availability of water in food systems. These factors directly influence the safety and behavior of starch during storage, since high values promote biochemical reactions and microbial growth [28,29]. Oca starches showed moisture contents below 11% and water activity (a_w_) values ranging from 0.40 to 0.44 (Table 1), with significant differences (*p* < 0.05). These values indicate sufficiently low water availability to inhibit microbial growth and ensure good storage stability [30,31,32]. The differences may be related to amylose content, as amylose’s linear structure has higher water affinity than amylopectin [33]. Accordingly, YOS, with the highest amylose content (24.71%), also showed slightly higher moisture.

On the other hand, microorganisms cannot grow at a_w_ < 0.60 [32], confirming the microbiological stability of these starches during storage. This observation aligns with polysaccharide-rich matrices, in which robust intermolecular hydrophilic interactions occur between starch hydroxyl groups (O–H) and water molecules, thereby reducing the amount of free water [31,34]. Variations among varieties are linked to differences in the amylose-to-amylopectin ratio (Table 2). Starches with higher amylopectin levels tend to hold more water within their granular structure due to their branched configuration, resulting in lower aw [20,34,35]. In contrast, starches with higher amylose content are more susceptible to releasing free water due to their propensity for easier association and retrogradation processes [20,34].

Zeta potential evaluates repulsive or attractive forces between starch granules, indicating suspension stability [36]. High values indicate greater electrostatic repulsion, preventing aggregation, while low values promote particle attraction and sedimentation [37]. Table 1 shows significant differences (*p* < 0.05) in zeta potential among varieties. WOS and BOS showed higher values (−29.29 mV and −25.25 mV, respectively), indicating moderate colloidal stability [38]. In contrast, YOS showed the lowest value (−14.39 mV), suggesting a less stable system prone to flocculation [38]. These differences are likely due to varietal factors, cultivation conditions, soil, climate, and agricultural practices affecting chemical composition [39]. These results suggest that WOS and BOS dispersions could remain relatively homogeneous for extended periods.

Particle size is a structural characteristic that influences physical, chemical, and functional properties, such as water absorption, swelling, gelatinization, and viscosity [20]. Significant differences (*p* < 0.05) were observed among varieties (Table 1). Larger particle sizes may be attributed to higher amylose content (Table 2), as amylose is directly related to granule growth [40]. Particle size also varies depending on botanical origin, variety, and growth conditions [24]. The distribution of starch granule size plays a decisive role in its functional and technological properties, as well as in the rate of hydrolysis when used as a raw material [41]. Granule size determines its potential applications, particularly in functions such as emulsification and encapsulation, and contributes significantly to the stability, texture, and functional performance of starch in different food and technological systems [42].

### 3.2. Amylose and Amylopectin Content

Table 2 presents the amylose/amylopectin ratio and shows significant differences among varieties. YOS had the highest amylose content (24.71%), while BOS had the lowest (21.81%). Amylose and amylopectin content significantly influence starch functional properties [30]. These obtained values are comparable to those reported for oca starch by other authors [9,43].

These differences are mainly due to genetic variability, botanical origin, harvest period, tuber type, cultural practices, and growth conditions, such as climate and soil type [20,44]. Likewise, granule particle size also directly influences structural composition [45]. Studies report that higher amylose content is associated with larger particles, consistent with the values in Table 1, whereas smaller granules are associated with lower amylose content [40,46]. This direct relationship impacts the structural and functional characteristics of starch [40]. The functional properties of starches are heavily influenced by their amylose and amylopectin levels [30]. High-amylose starches exhibit strong gelling properties, making them useful in pasta, confectionery, bread, and coating applications [47].

### 3.3. Morphological Analysis

**Figure 2:**
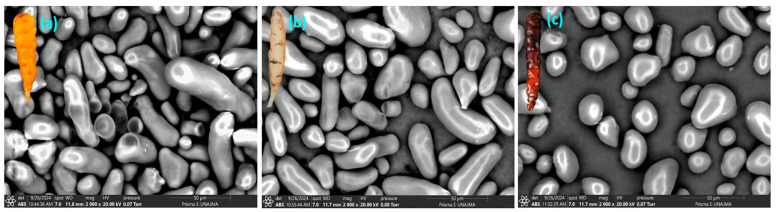
(**a**) SEM image of YOS, (**b**) SEM image of WOS, and (**c**) SEM image of BOS.

Scanning Electron Microscopy (SEM) is essential for analyzing the microstructure of starch granules, providing detailed information on morphology, size, and surface characteristics [48]. SEM micrographs revealed morphological differences among varieties. YOS and WOS granules exhibited oval, elliptical, and elongated shapes [49,50,51], while BOS granules were spherical and oval [39,52]. Granules were dispersed within the starch matrix, showing morphological variability. These shapes are typical of tuber starches, especially those from Andean roots and tubers, where amyloplast development results in heterogeneous granule geometry [53,54]. All starches showed smooth, planar surfaces without fissures, indicating native, structurally intact granules [55]. As shown in Figure 2, shape differences among isolated starches are due to botanical source and variety [51].

### 3.4. XRD and IR Analysis

**Figure 3:**
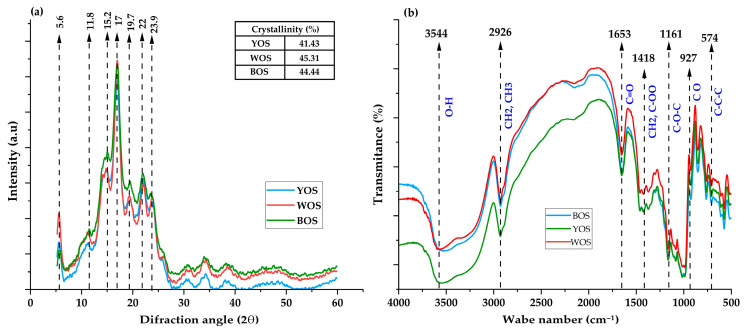
(**a**) X-ray diffractogram and degree of crystallinity; (**b**) FTIR spectra of oca starches of three varieties.

Figure 3 integrates the XRD and FTIR results for WOS, BOS, and YOS samples. The techniques characterize the semicrystalline organization and functional groups of the polymer, providing essential structural and behavioral information.

XRD was used to determine the starch crystalline pattern and quantify crystallinity. The XRD patterns (Figure 3a) show a B-type crystallinity pattern, characterized by defined peaks at 2θ angles of 5.6, 11.8, 15.2, 17, 19.7, 22, and 23.9°, consistent with reports for Andean tuber starches [17,56,57]. These peaks result from the semicrystalline structure of the starch, where crystalline and amorphous regions coexist. B-type patterns appear when amylopectin double helices assemble into a highly hydrated hexagonal structure with wide cavities that allow the incorporation of water molecules [34,57,58,59]. The most intense peak at 2θ ≈ 17° is the main marker of the B-pattern, indicating a high degree of amylopectin double-helix ordering within crystalline regions [60,61]. The greater intensity of this peak in WOS and BOS samples suggests a more ordered structure compared to YOS.

A higher degree of crystallinity was reported for WOS and BOS (45.31% and 44.44%, respectively), indicating a greater proportion of crystalline regions, while YOS showed 41.32%. These values are comparable to those reported by other authors [17,61,62]. Variation may be influenced by variety, cultivation conditions, and isolation method [34,63].

FTIR is widely used to characterize the chemical structure of starch. Spectra in the 400–4000 cm^−1^ range are shown in Figure 3b. A broad band around 3500 cm^−1^ indicates the presence of hydroxyl (-OH) groups in glucose chains [64,65]. These absorptions correspond to both the stretching and bending vibrations of the bonds present in these groups, which are fundamental components of amylose and amylopectin molecules. These absorption bands are comparable to those reported by other researchers [17].

Similarly, a characteristic band around 2926 cm^−1^ is observed, due to C-H bond stretching vibrations present in the D-glucopyranose structure, the starch monomeric unit. Although associated with CH_2_ and CH_3_ groups [66], the principal contribution originates from methylene groups (CH_2_), as methyl groups (CH_3_) are less abundant in starch structures. Vibrations at 1653 cm^−1^ indicate the presence of a carbonyl group [17]. Several factors, such as protein presence, polysaccharide structure, and adsorbed water quantity, influence C=O bond vibration in starch. Hydration plays a key role in C=O band displacement, reflecting hydrogen-bond interactions in starch’s amorphous region. The peak at 1418 cm^−1^ is associated with bending vibrations of methylene groups (CH_2_) and carboxylate groups (COO^−^) and carbohydrates [17,67,68], providing information on polysaccharide structure and modifications, such as starch [69].

Another band around 1161 cm^−1^ is observed, corresponding to asymmetric C-O-C stretching vibrations in the glucopyranose ring [17,70] and glycosidic bonds connecting glucose units in starch polymers [17,67]. The 927 cm^−1^ peak is attributed to C-O stretching vibrations, particularly important for identifying α-1,4 bonds, which form the principal structure of amylose and amylopectin [17,64,67]. The 580 cm^−1^ peak corresponds to C-C-C bond bending vibrations in starch glycosidic bonds [17,68]. These results coincide with the crystallinity obtained by XRD (Figure 3a), where WOS shows the highest crystallinity (45.31%), followed by BOS and YOS. According to the literature, more crystalline type-B starches show greater intensity in FTIR bands associated with helical order and greater structural water content, which agrees with the observed patterns.

### 3.5. Swelling Power (SP), Water Absorption Capacity (WAC), and Water Solubility Index (WSI)

Techno-functional properties are crucial for industrial and domestic starch use, describing the relationship between water molecules and starch polymer chains in semicrystalline regions under temperature changes [57,71].

**Figure 4:**
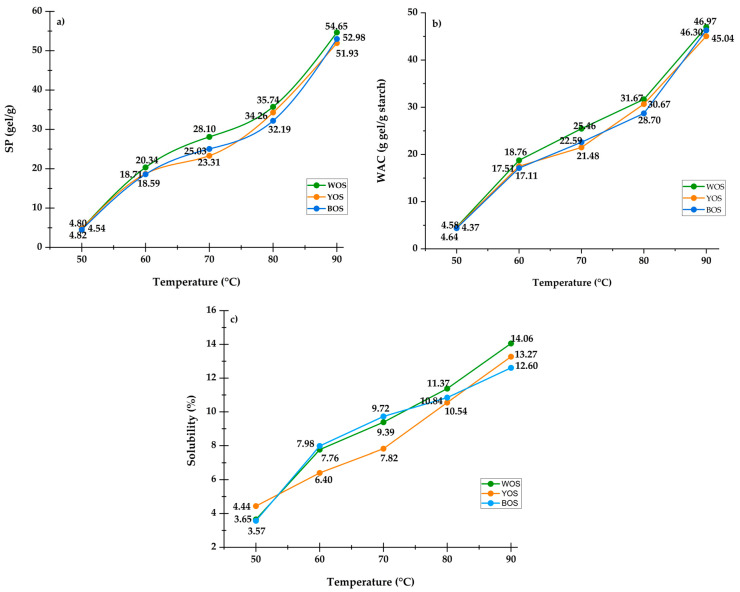
Techno-functional properties of oca starch of three varieties at different temperatures. (**a**) Swelling power, (**b**) water absorption capacity, and (**c**) solubility.

The SP, WAC, and WSI for oca starches are shown in Figure 4a–c. SP reflects the capacity of starch granules to absorb water and expand when heated above the gelatinization temperature. Figure 4a shows that SP increased from 4.80 to 51.94 g gel/g for YOS, 4.82 to 54.66 g gel/g for WOS, and 4.54 to 52.99 g gel/g for BOS in the 50–90 °C range, showing a sustained increase as gelatinization progresses. Variation in SP among samples is primarily associated with amylose/amylopectin ratios (Table 2). Higher amylopectin content favors water absorption and granule expansion due to its branched, flexible structure, enhancing hydration and thermal disorganization. Studies report that starches with higher amylopectin tend to exhibit greater swelling power [17,72,73,74].

Similarly, WAC indicates the amount of water starch can absorb as the temperature increases [75]. Figure 4b shows that WAC progressively increased between 50 and 90 °C, reaching values from 4.38 to 46.98 g gel/g. This behavior is influenced by granule size (Table 1) and the presence of hydrophilic groups (Figure 2b). Smaller granules, with a higher amylopectin proportion, exhibit greater water retention than larger granules typical of amylose-rich structures. In addition, the number of hydrophilic groups in the starch matrix also significantly contributes to increased water absorption at elevated temperatures [30,76,77].

On the other hand, WSI indicates the ability of starch solids to disperse in aqueous solution during swelling while the temperature increases [78]. Figure 4c shows that WSI increased progressively with temperature from 50 to 90 °C, ranging from 4.44 to 13.27% for YOS, 3.65 to 14.06% for WOS, and 3.57 to 12.61% for BOS. These differences are due to the lower amylose content in oca starches (Table 2), as high-amylose starches have greater solubility because amylose solubilizes and migrates out of the granule during swelling [79]. This study demonstrated that amylopectin content is related to swelling power, water absorption capacity, and solubility index. High-quality starches exhibit high absorption capacity, low solubility, and high swelling power [80].

Based on the obtained results, the starch shows high potential for application as a thickening, binding, gelling, and stabilizing agent in food systems, due to its relatively high pH values and high water absorption capacity (WAC), properties that promote the formation of viscous and stable structures in food matrices [20,81].

### 3.6. Rheological Analysis

The rheological properties of oca starches were analyzed through their relationship with shear stress (τ) and apparent viscosity (μ) versus shear rate (γ), establishing the flow type of the system.

**Figure 5:**
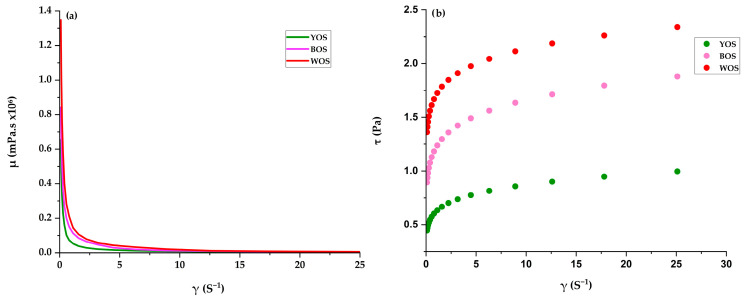
Rheological analysis of three varieties of oca starch: (**a**) viscosity vs. shear speed; (**b**) shear stress vs. shear speed.

Apparent viscosity (Figure 5a) shows that oca starches exhibit pseudoplastic behavior, with viscosity decreasing as deformation rate increases. This pseudoplasticity is due to the progressive alignment and disordering of amylose and amylopectin chains under shear [82,83]. The higher viscosity of WOS compared to BOS and YOS may be attributed to its higher amylopectin content (Table 2), as this branched component favors the formation of more rigid molecular networks resistant to flow [82]. In contrast, the lower viscosity of WOS and YOS may be explained by higher amylose availability (Table 2), higher crystallinity, greater internal granule cohesion, or differences in granule structure that affect swelling capacity [17]. According to [83], variations in composition and granule size generate significant viscosity differences among starches.

Shear stress (Figure 5b) increased with shear rate for all samples, indicating pseudoplastic non-Newtonian behavior due to progressive disorganization of the granular structure under shear [83,84]. WOS showed the highest shear stress, indicating a more flow-resistant structure, as differences in granular integrity and molecular distribution generate variations in mechanical resistance during shear [85].

### 3.7. Thermal Stability

**Figure 6:**
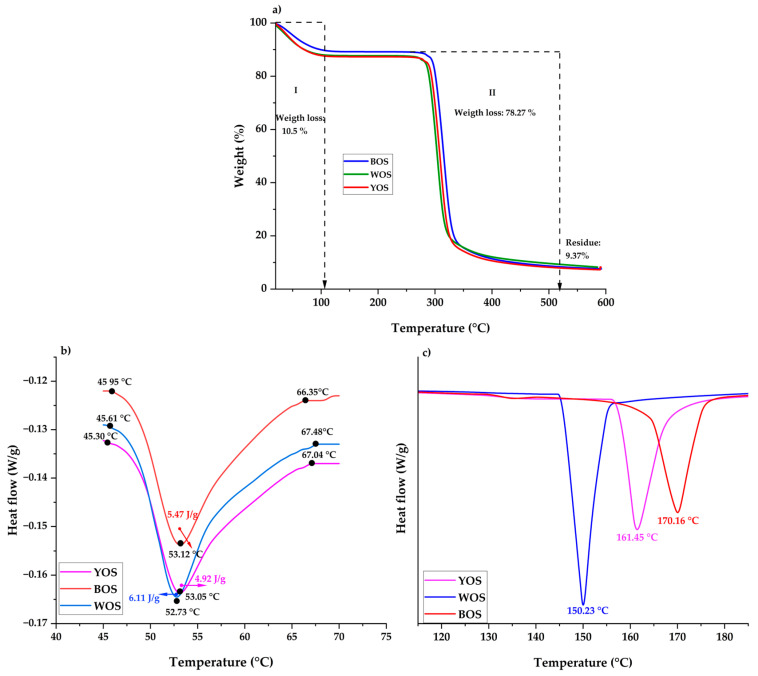
TGA and DSC thermograms of oca starch of three varieties in terms of (**a**) loss of mass; (**b**) gelatinization temperature; and (**c**) melting temperature.

Starch mass loss was evaluated by Thermogravimetric Analysis (TGA), which showed two principal degradation stages below 600 °C (Figure 6a). In the first zone (first drop), initial starch mass loss occurred around 10.5% across a temperature range of 20 to 110.25 °C. This loss corresponds mainly to the elimination of free and weakly bound water molecules present in starch [86,87]. These molecules are released at relatively low temperatures because they maintain weak physical interactions with the polymer matrix [88]. Furthermore, mass remains constant from 110.25 °C to 265.45 °C, indicating that complex high-molecular-weight structures such as polysaccharides (amylose and amylopectin) continue to be present in starches without experiencing degradation, preserving their thermal integrity, as glycosidic bonds present stability in this interval.

Subsequently, a second zone (second drop) is observed, representing the major decomposition phase, with a mass loss of 78.27% over a temperature range of 265.45 °C to 525.65 °C. This stage undergoes major decomposition of the predominant compounds present in starch samples, such as carbohydrates (amylose and amylopectin). This decomposition initiates through rupture of α-(1 → 4) and α-(1 → 6) glycosidic bonds, followed by depolymerization and protein denaturation [89]. Above 525.65 °C, mass remained constant, leaving 9.37% as residue (ash) for the yellow, white, and black oca starches, respectively.

Differential Scanning Calorimetry (DSC) is fundamental for characterizing starch gelatinization, which is influenced by factors such as amylose/amylopectin content, granule size, crystallinity, and water interactions [34,35]. The thermograms in Figure 6b show changes during oca starch gelatinization. Onset gelatinization temperature (To) ranged from 45.30 to 45.95 °C, peak temperature (Tp) from 52.73 to 53.12 °C, and conclusion temperature (Tf) from 66.55 to 67.48 °C. Gelatinization enthalpy varied from 4.92 to 6.11 J/g. These values indicate lower gelatinization temperatures than those reported for other oca starches, with T0 49.5 °C, Tp 54.6 °C, and Tf 61.4 °C [90]. Potato starch showed T0 from 49.31 to 54.17 °C, Tp from 54.69 to 59.75 °C, and Tf from 60 to 66.28 °C, with an enthalpy range of 3.60 to 6.62 J/g [17].

On the other hand, gelatinization enthalpy (ΔH) is directly related to crystal concentration, reflecting progressive loss of molecular order [79]. Lower enthalpy indicates less energy required to initiate gelatinization, which may be associated with lower structural stability or lower granule crystallinity [91]. Enthalpy is also influenced by granule morphology and size [92,93,94].

Figure 6c shows an endothermic peak in the range of 150.23 to 170.16 °C, associated with the dissociation of amylose–lipid complexes or the melting of recrystallized amylose structures formed during retrogradation [95]. These structures exhibit a higher degree of order and thermal stability, and therefore require higher temperatures for their disruption. Furthermore, this behavior may be influenced by the amylose/amylopectin ratio and by the presence of components such as lipids or proteins, which promote the formation of more thermally stable structures [96,97]. Higher melting temperatures suggest greater thermal stability, whereas lower melting temperatures may indicate greater resistance to thermal decomposition [20,34].

## 4. Conclusions

The study of starch from three oca varieties enabled characterization of their properties, revealing significant differences among them. White oca starch (WOS) showed the highest water absorption capacity (46.98 g gel/g) and swelling power (54.66 g gel/g), suggesting higher amylopectin content (78.14%). In contrast, yellow oca starch (YOS) exhibited the highest amylose content (24.71%), resulting in lower swelling and solubility capacities. FTIR analysis confirmed the presence of characteristic polysaccharide functional groups in all evaluated starches, with variations in the intensities of the hydroxyl and carbonyl bands among varieties. SEM revealed oval to spherical starch granules with smooth surfaces; the white variety (WOS) exhibited the largest granules. Rheologically, all three varieties exhibited typical pseudoplastic behavior of non-Newtonian systems. XRD analysis revealed a B-type crystallinity pattern in all cases, characteristic of starches with more hydrated structures and less dense packing.

Overall, these results indicate that oca starches, particularly those from the white variety, exhibit functional properties suitable for specific applications in the food industry, such as thickening and texturizing agents in beverages, sauces, and gelled products. These findings contribute to the revalorization of oca as a strategic agroindustrial resource and support its incorporation into value chains with greater technological and economic impact.

## Figures and Tables

**Table 1:** Yield, color, whiteness index, humidity, water activity, zeta potential, and particle size of starches.

Parameters	YOS			WOS			BOS		
	$\bar{x}$	±	S	$\bar{x}$	±	S	$\bar{x}$	±	S
Yield (%)	7.58 ^a^	±	0.12	8.25 ^b^	±	0.12	7.79 ^c^	±	0.06
L*	93.83 ^a^	±	0.05	94.63 ^b^	±	0.04	92.28 ^c^	±	0.08
a*	0.06 ^a^	±	0.00	0.27 ^b^	±	0.01	−0.04 ^c^	±	0.00
b*	1.27 ^a^	±	0.01	1.16 ^b^	±	0.01	1.71 ^c^	±	0.01
WI	93.7 ^a^	±	0.05	94.5 ^b^	±	0.03	92.1 ^c^	±	0.08
YI	1.93 ^a^	±	0.02	1.75 ^b^	±	0.02	2.65 ^c^	±	0.02
CI	0.51 ^a^	±	0.00	2.43 ^b^	±	0.06	−0.25 ^c^	±	0.00
a_w_	0.44 ^a^	±	0.00	0.40 ^b^	±	0.00	0.43 ^c^	±	0.00
Moisture (%)	9.57 ^a^	±	0.08	10.02 ^b^	±	0.04	9.36 ^a^	±	0.14
Zeta potential (mV)	−14.30 ^a^	±	0.09	−29.29 ^b^	±	0.43	−25.25 ^c^	±	0.25
Particle size (µm)	27.74 ^a^	±	0.05	27.56 ^b^	±	0.05	26.32 ^c^	±	0.04

The values expressed are the mean ± standard deviation. Data with different letters in the same column differ statistically significantly (*p* < 0.05).

**Table 2:** Amylose and amylopectin content.

Parameters	YOS			WOS			BOS		
	$\bar{x}$	±	S	$\bar{x}$	±	S	$\bar{x}$	±	S
Amylose	24.71 ^a^	±	0.11	22.86 ^b^	±	0.04	21.81 ^c^	±	0.08
Amylopectin	75.29 ^a^	±	0.11	78.14 ^b^	±	0.04	77.19 ^c^	±	0.08

The values expressed are the mean ± standard deviation. Data with different letters in the same column differ statistically significantly (*p* < 0.05).

## Data Availability

The original contributions presented in this study are included in the article. Further inquiries can be directed to the corresponding authors.
